# Medical students’ perceptions of LGBTQ+ healthcare in Singapore and the United Kingdom

**DOI:** 10.3389/fmed.2023.1236715

**Published:** 2023-10-24

**Authors:** Michael X. Fu, Tangming Zou, Raksha Aiyappan, Xinyu Ye, Simisola Onanuga, Angela Tan, Susan Smith, Ana Baptista

**Affiliations:** ^1^Medical Education Research Unit, Imperial College London, London, United Kingdom; ^2^Lee Kong Chian School of Medicine, Nanyang Technological University, Singapore, Singapore

**Keywords:** LGBTQ+, medical education, curriculum, attitudes, bias training

## Abstract

**Introduction:**

Lesbian, gay, bisexual, transgender, queer, and other sexual and gender minority (LGBTQ+) individuals have an increased scope of healthcare needs and face many barriers to accessing healthcare. However, LGBTQ+ healthcare education remains scarce, and students’ understanding of LGBTQ+ healthcare remains largely uncharacterised. This study investigated the knowledge of and attitudes toward LGBTQ+ healthcare among medical students in Singapore and the United Kingdom (UK), two culturally different countries.

**Methods:**

Medical students in two medical schools, one in Singapore and the other in the UK, completed self-administered cross-sectional surveys using multiple-choice, Likert scale, and free-text questions to explore their ideas, concerns, and expectations about LGBTQ+ healthcare education within their medical curricula.

**Results:**

From 330 responses, students’ knowledge levels were moderate overall, with pronounced gaps in certain areas, including terminology, sexual health, and conversion therapy. Deficiencies in knowledge were significantly greater among students in Singapore compared to the UK (*p* < 0.001), whilst LGBTQ+ students and non-religious students had more positive knowledge and attitudes than students not identifying. At least 78% of students had positive attitudes towards LGBTQ+ individuals, but 84% had not received LGBTQ+-specific medical education. Although junior UK students were more satisfied with the adequacy of teaching by their medical school’s incorporation of LGBTQ+ inclusive teaching in a newer curriculum, qualitative analyses suggested that students in both countries wanted to receive more training. Students further suggested improvements to the medical curriculum to meet their needs.

**Conclusion:**

Students in both schools lacked understanding of commonly-used terminology and topics such as sexual healthcare despite affirming attitudes towards LGBTQ+ healthcare. Although sociolegal contexts may affect students’ perspectives, differences were less than thought, and students were equally keen to provide affirmative care to their patients. They emphasised a need for more formal teaching of LGBTQ+ healthcare professions to overcome healthcare disparities in these communities.

## 1. Introduction

Lesbian, gay, bisexual, transgender, queer, and other sexual and gender minority (LGBTQ+) persons constitute marginalised groups in society that face individual and systematic stigmatisation (1) and difficulties in accessing healthcare (2). Evidence suggests LGBTQ+ individuals may experience health disparities in a variety of domains compared to non-LGBTQ+ individuals (3, 4), such as the high burden of mental health problems encountered in LGBTQ+ populations; for example, the governmental United Kingdom LGBT survey concluded that 24% of respondents had accessed mental health services in a 12-month period (5), compared to 4.5% of all individuals in England in a similar period (6). The heteronormative endemic in society may contribute to the inaccessibility of healthcare for LGBTQ+ individuals, where society’s heteronormative social order may subconsciously dictate medical interactions and act as a barrier to access to vital social institutions for LGBTQ+ individuals (7). Heteronormative beliefs at the point of care have also been attributed to discrimination of LGBTQ+ individuals by doctors, exacerbated by doctors’ lack of knowledge and low self-efficacy in interacting with LGBTQ+ patients (8, 9). Educating healthcare professionals about LGBTQ+ healthcare is considered the most effective way to improve engagement with patients (10), prompting discussions to include diversity-related competencies in medical curricula (11), where medical curricula lack such content at present (12–14). It has been shown that LGBTQ+-focused educational programmes improve students’ attitudes, knowledge, and comfort levels (9). However, it is essential to consider students’ perspectives and motivation for change when planning educational interventions to reduce participant bias (9).

Limited research exists on students’ perspectives regarding LGBTQ+ healthcare. American students expressed comfort in interacting with LGBTQ+ patients but felt a lack of formal education (15). Despite very recent calls from the British Medical Association for teaching and learning about LGBTQ+ healthcare needs in medical education without stereotypes (16), British students had low confidence in using sexual and gender terminology (17, 18). European surveys showed that knowledge and attitudes might depend partly on socio-demographic factors, including respondents’ gender identification and religiosity (19, 20).

Singapore is an English-speaking country located in Southeast Asia that, until November 2022, retained male homosexual criminalisation inherited from British colonisation (21). Though Singaporean society may be growing in acceptance of the LGBTQ+ communities, widespread religious resistance and stigma still exist (21), where 57% of Singaporean society remains opposed to homosexuality compared to 13% of British society (22). Same-sex partnerships remain unrecognised in Singapore, with public policies that fail to affirm LGBTQ+ individuals in the workplace and housing (23). 60.2% of the LGBTQ+ community in Singapore have experienced abuse and discrimination regarding their sexuality and gender identity, exacerbating mental health issues (24). Gender-affirming care is reported to be challenging to access amid stigma and insufficient institutional and social support (25).

To the best of our knowledge, no study has characterised healthcare professionals’ or future doctors’ perceptions of the LGBTQ+ communities in Singapore. Though LGBTQ+ individuals in the United Kingdom also face discrimination, the social, cultural, and legal environment differs significantly between the United Kingdom and Singapore. LGBTQ+ issues are divisive across generational lines (26), so it is instructive to investigate how young people experience a curriculum created by an older generation, particularly in one country where, at the time this study was carried out, male homosexuality was illegal. This study compares how medical students perceive LGBTQ+ healthcare in a country where homosexuality was decriminalised more than 50 years ago, one where it was decriminalised only very recently, and where societal attitudes differ markedly in the two countries. Uniquely, the two medical schools in this study have similarly structured curricula and studies between medical students from both schools are actively encouraged and funded.

## 2. Materials and methods

### 2.1. Instrument development

The authors considered existing surveys at the time of survey design (27, 28) to contain stigmatising language towards the LGBTQ+ communities. We developed a new survey with more inclusive language and free-text components absent from previous surveys (29). The MEDLINE database was searched to identify relevant health needs, facilitated by discussions between the co-investigators, medical education experts, and a representative from an LGBTQ+ non-profit community organisation. The survey was piloted with members of an LGBTQ+-identifying healthcare staff network in London. Their responses were compared with students’ responses to the factual questions. The entire survey was further piloted with six medical students to check for accuracy, flow, and understanding, and their suggestions were incorporated into the final version. The internal consistency of each survey section was measured using the alpha coefficient Kuder–Richardson 20 (KR-20) for dichotomous data or Cronbach’s alpha for non-dichotomous measures in SPSS (version 28.0.0, IBM).

The survey consisted of socio-demographic questions, multiple-choice knowledge questions surrounding agreed-upon terminology to refer to people who identify in a particular way, and language commonly used in healthcare for LGBTQ+ individuals. Further questions probed students’ knowledge of gender transitioning, blood donation restrictions, the prevalence of HIV/AIDS, HIV medication, whether homosexuality is a psychological condition and understanding of gender dysphoria. Students were also given clinical scenarios to assess whether they would ask for patients’ preferred pronouns and what they would do for patients seeking conversion therapy. Care was taken to include a mixture of more general and specific knowledge questions, as judged by the expert panel developing the items. Attitudes and approval toward specific healthcare practices such as using gender-neutral pronouns, asking for consent before recording gender and sexual orientation, and providing affirming care were assessed on a 5-point Likert scale. Students were then asked to rate their learning regarding LGBTQ+ patients in various settings on a Likert scale and were asked about their sources of knowledge on LGBTQ+ issues and specific topics. Open-ended questions in the final section of the survey asked about difficulties faced when trying to learn more about LGBTQ+ patients, topics students wanted to learn more about, and changes they wanted to see in formal teaching regarding LGBTQ+ health issues. We utilised a pragmatist philosophical approach underpinned by realist ontology, using both a quantitative approach to gather real-world perspectives and a qualitative approach to investigate students’ reasons and needs behind these perspectives (30). Ethics approval was obtained from the Imperial College Research Ethics Committee (21IC7342) and the Nanyang Technological University Institutional Review Board (IRB-2021-521).

### 2.2. Procedures

Respondents were recruited via student newsletters and year-wide social media groups, inviting voluntary participation at a time selected to avoid survey fatigue and examination periods. Respondents self-completed the online Verint survey on their own devices and in their own time. Consent was given on an introductory page. A prize draw incentive for GBP£10/SGD$20 vouchers directed survey participants to a separate form to retain survey data anonymity. The complete survey is available as Supplement 1.

### 2.3. Participants

Inclusion criteria allowed medical students 18 years or older to participate during the academic year 2021–2022, registered at one of two specific medical schools, one in London (UK) and the other in Singapore (SG). At the time of the study, these medical schools were partnered with similar curricula [descriptions of curricula found on respective websites (31, 32)]. The SG medical school has teaching on delineating sex and gender. The UK medical school was implementing curriculum change at the time of the study, commencing with year 1 students who started in the academic year 2019–2020. From personal communications with faculty in 2023 and scrutinising the published learning outcomes in both curricula, the older curriculum had outcomes related to health inequalities and discriminatory practices but did not explicitly separate LGBTQ+ content. It is difficult to know how individual educators addressed these learning outcomes. The newer curriculum incorporates sensitivity training from Year 1 that explicitly includes hypothetical scenarios with gay and trans people, recognising heteronormative assumptions in clinical communications, teaching on working sensitively with gender and sexually diverse groups in Year 2 and patient cases with LGBTQ+ couples in Years 2 & 3. Teaching about gender as a social construct and the difference between gender and sex is also explicitly included in the newer curriculum. It is noted that certain aspects of the newer curriculum may not have been introduced at the time of this study. Students in years 1 to 3 in the academic year 2021–2022 received the newer curriculum, while students in years 4 to 6 received the older curriculum.

### 2.4. Data analysis

Quantitative data analyses were conducted in GraphPad Prism (version 9.5.0, LLC). Analyses were conducted overall, comparing medical schools, junior (years 1 to 3) and senior (years 4 to 6) years, LGBTQ+ identification, gender identification, and religiosity. Since no respondents identified as “other gender” (Table 1), and with the small number of non-binary respondents solely in the UK insufficient for statistical analysis, analyses were performed between respondents who self-identified as male and female, as in previous studies (33, 34). Further analyses were conducted between junior and senior UK students to assess the effect of changes in curricula on responses. Normality was assessed using the Shapiro-Wilks test. For knowledge questions, the Mann–Whitney U test compared between groups, with data shown as the median (interquartile range). Categorical data were assessed with Fisher’s exact test and Chi-squared test. To reduce the variability from subjectiveness, responses from Likert scale data were grouped into positive and negative responses before statistical analyses, omitting neutral responses. The significance level for all inferential analyses was set at *p* ≤ 0.05.

**Table 1:** Demographics of respondents with comparisons between the United Kingdom medical school (UK) and Singapore medical school (SG).

Demographic	UK (*n* = 151)	SG (*n* = 179)	*p*-value	All (*n* = 330)
Age (median, IQR)	21 (20–22)	21 (20–22)	0.395	21 (20–22)
Year of study, *n* (% of total UK/SG/all respondents)				
Year 1	9 (6%)	33 (18%)	–	42 (13%)
Year 2	18 (12%)	50 (28%)		68 (21%)
Year 3	31 (21%)	31 (17%)		62 (19%)
Year 5 (UK)/Year 4 (SG)	30 (20%)	30 (17%)		72 (22%)
Year 6 (UK)/Year 5 (SG)	21 (14%)	35 (20%)		75 (23%)
Year 4 (UK)	42 (28%)	-		21 (6%)
Junior students*	*58 (38%)*	*114 (64%)*	<0.001^+^	*172 (52%)*
Senior students*	*93 (62%)*	*65 (36%)*		*158 (48%)*
Gender identification, *n* (%)				
Male	51 (34%)	94 (53%)	0.002^=^	145 (44%)
Female	94 (62%)	83 (46%)		177 (54%)
Non-binary	4 (3%)	0 (0%)		4 (1%)
Other gender	0 (0%)	0 (0%)		–
Prefer not to say	2 (1%)	2 (1%)		4 (1%)
Respondents identifying as LGBTQ+, *n* (%)				
Lesbian	7 (5%)	0 (0%)	–	7 (2%)
Gay	18 (12%)	8 (4%)		26 (8%)
Bisexual	36 (24%)	15 (8%)		51 (15%)
Transgender	3 (2%)	0 (0%)		3 (1%)
Queer	8 (5%)	0 (0%)		8 (2%)
Other LGBTQ+identity	1 (1%)	1 (1%)		2 (1%)
*Total*	*73 (48%)*	*24 (13%)*	<0.001^+^	*97 (29%)*
Religious identification, *n* (%)				
Identify with religion	61 (40%)	108 (60%)	<0.001^=^	169 (51%)
No religion	73 (48%)	67 (37%)		140 (42%)
Prefer not to say	17 (11%)	4 (2%)		21 (6%)

*UK junior students received the newer curriculum, whilst UK senior students received the older curriculum, ^+^: Fisher’s exact test, ^=^: Chi-squared test. “%” represents the percentage of UK, SG, and all respondents (in their respective columns) who indicated the demographic option. IQR = interquartile range, *n* = the number of respondents, LGBTQ+ = lesbian, gay, bisexual, transgender, queer, and other.

For the open-ended questions, thematic analysis was performed on NVivo (release 1.6.2, QSR International). Five investigators performed an iterative process of generating themes, at first very concrete and representative of the data, but which became more conceptual with repetitive rounds of discussion and coding (35, 36). Discrete ideas were discussed collaboratively between the investigators, where one response may have more than one discrete concept. Iterative review processes enabled the categorisation of these ideas into succinct codes. As more data were re-reviewed, codes were also revised and grouped. After coding all data, the codes were further reviewed and grouped into higher-level themes. Other investigators audited the final coding.

## 3. Results

### 3.1. Instrument piloting

The question about the proportion of LGBTQ+ individuals in each country was removed from the analysis since it is difficult to ascertain their true proportions, leaving twelve questions in the knowledge section. Compared with all students’ responses for the knowledge section (median score 10 [8–11]; maximum score possible being 12/12), the expert group of 25 LGBTQ+ respondents scored significantly higher (12 [11–12], *p* < 0.001), demonstrating face validity. Statistical analysis of knowledge-based questions gave a KR-20 score of 0.59, perhaps unsurprisingly, since this section encompassed many unrelated facets of knowledge ranging from terminology to clinical scenarios. Non-dichotomous survey sections showed good levels of internal consistency with Cronbach’s alpha of 0.81 in both the attitudes and sources of knowledge sections.

### 3.2. Demographics

346 respondents completed the survey. 16 respondents had already graduated and did not meet inclusion criteria, leaving 330 included entries, consisting of 151 UK and 179 SG respondents (Table 1) from approximately 1,500 and 700 eligible students in UK and SG, respectively. There were similar numbers of junior and senior students. 29% of all respondents identified as LGBTQ+, with 48% of UK respondents compared to 13% of SG respondents (*p* < 0.001). There was a similar proportion of LGBTQ+ students between junior and senior UK students (52% vs. 40% respectively, *p* = 0.290). 51% of all respondents identified with a religion, with a more significant proportion from SG than the UK (60% vs. 40%, *p* < 0.001).

### 3.3. LGBTQ+ healthcare knowledge

**Figure 1:**
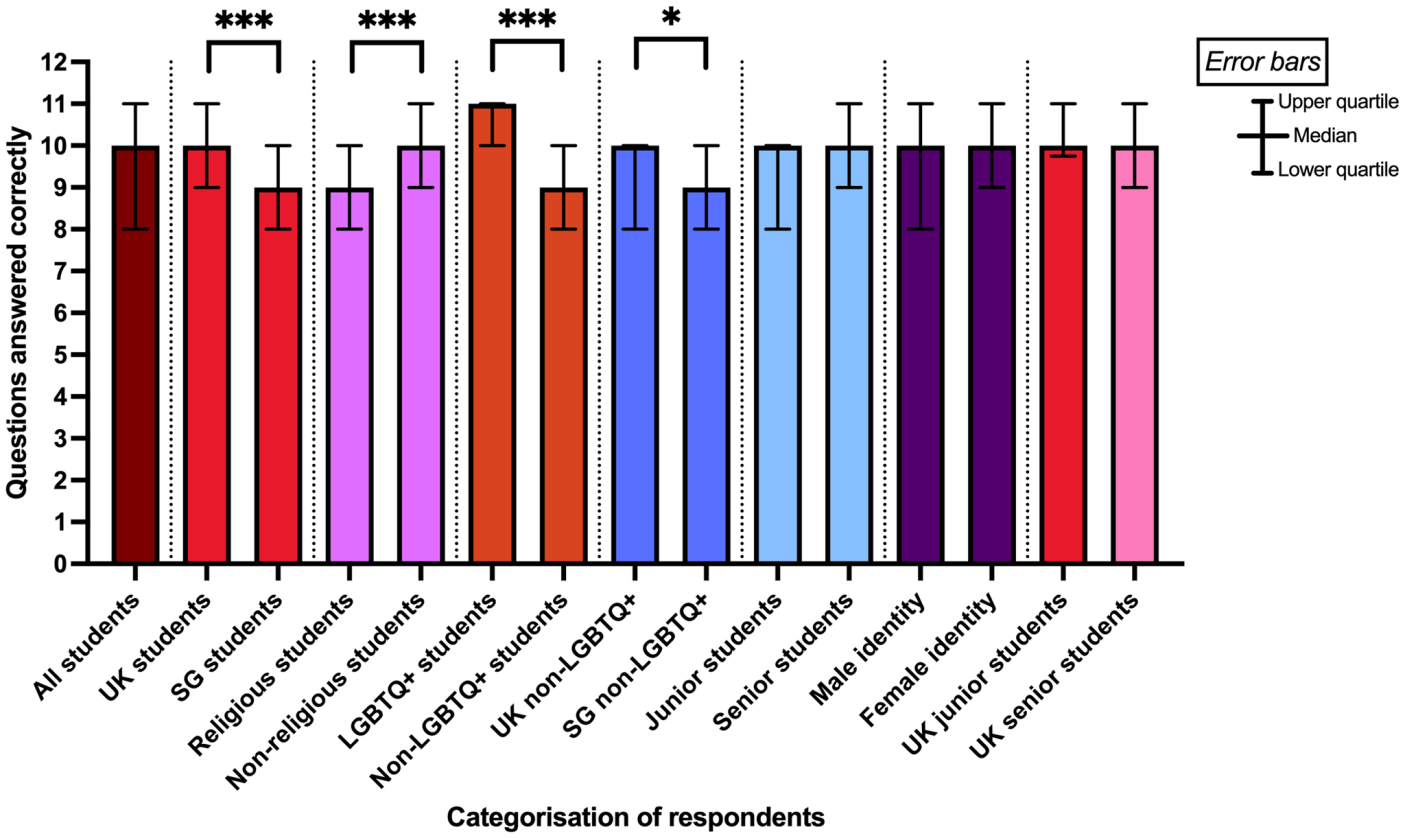
Comparisons of questions answered correctly from the Knowledge section of the survey between pairs of groups of respondents. * denotes *p <* 0.05, *** denotes *p* < 0.001 from Mann–Whitney U tests. The data were non-normal, where the bars in the figure show median values, and the error bars represent interquartile ranges.

**Figure 2:**
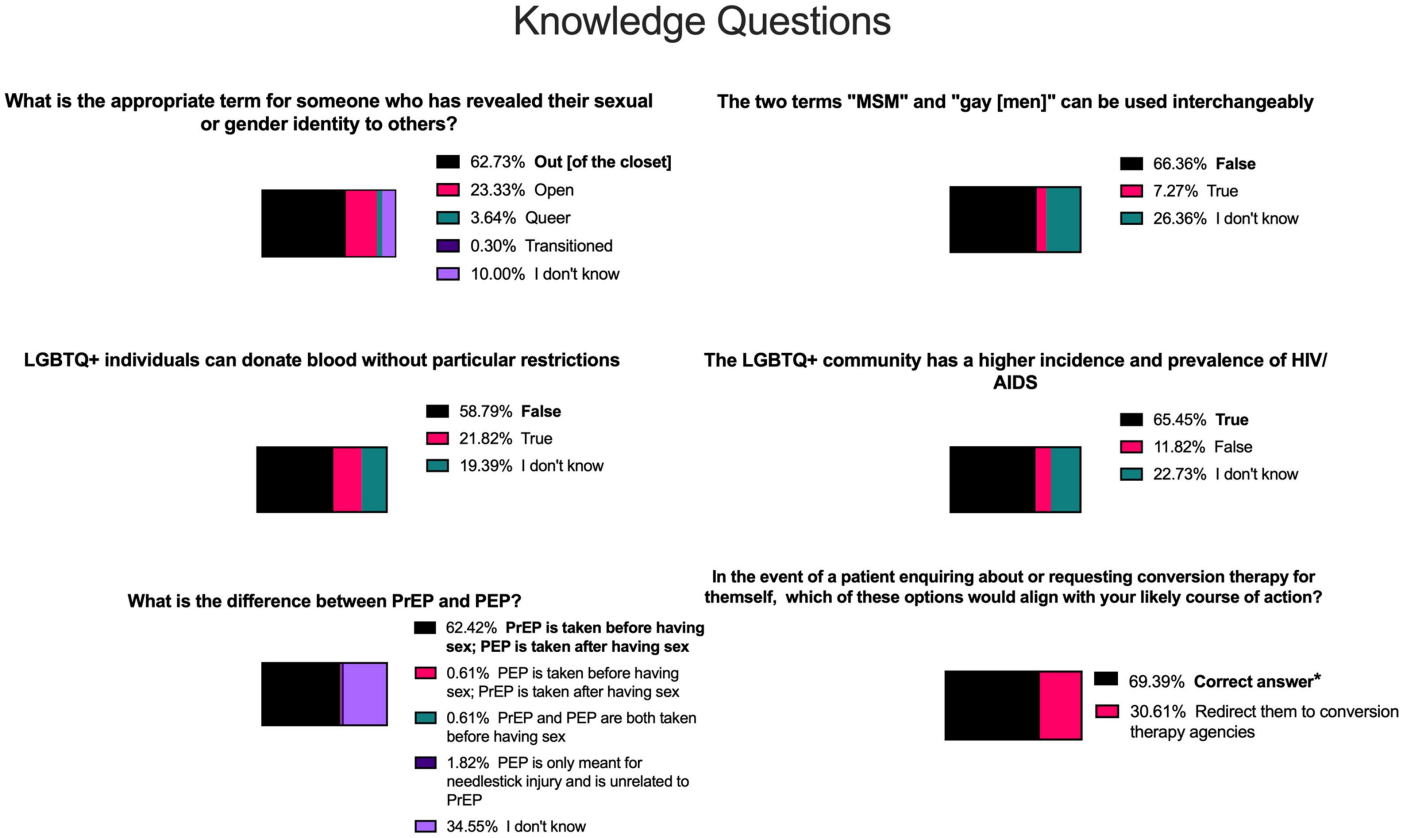
Knowledge-based questions most frequently answered incorrectly. Statements highlighted in bold and shaded in black show the correct answer for each question. Other shades of colours indicate incorrect answers or where students indicated “I do not know”. *A correct answer consisted of one or more of the following answers: “Explain to them that conversion therapy does not have enough scientific evidence to support it”, “Assist your patient in understanding more about various sexual orientations”, and “Redirect them to organisations providing support services for LGBTQ+ individuals”, without selection of the incorrect response “Redirect them to conversion therapy agencies”. MSM = men who have sex with men, LGBTQ+ = lesbian, gay, bisexual, transgender, queer, and other, HIV = human immunodeficiency virus, AIDS = acquired immune deficiency syndrome, PrEP = pre-exposure prophylaxis, PEP = post-exposure prophylaxis.

Of the 12 knowledge-based questions, respondents correctly answered an average of 10 [8–11]. UK students correctly answered slightly more questions than SG students (10 [9–11] vs. 9 [8–10] respectively; *p* < 0.001; Figure 1). Non-religious students answered slightly more correctly than religious students (9 [9–11] vs. 9 [8–10], respectively; *p* < 0.001) and LGBTQ+ individuals also answered significantly more correctly than non-LGBTQ+ respondents (11 [10–11] vs. 9 [8–10], respectively; *p* < 0.001). Considering that the expert group answered better than the undifferentiated population of students and the potential skew of results from the higher proportion of LGBTQ+ respondents in the UK than in SG, we compared the knowledge of non-LGBTQ+ students between the UK and SG; UK respondents still answered significantly better than their SG counterparts (10 [8–10] vs. 9 [8–10], respectively; *p* = 0.049). There were no differences when comparing junior and senior students (*p* = 0.248), including in the UK (*p* = 0.717), or between respondents who self-identified as male and female (*p* = 0.277). Considering specific questions, six questions had >30% of incorrect responses (Figure 2).

### 3.4. Attitudes towards LGBTQ+ healthcare

**Figure 3:**
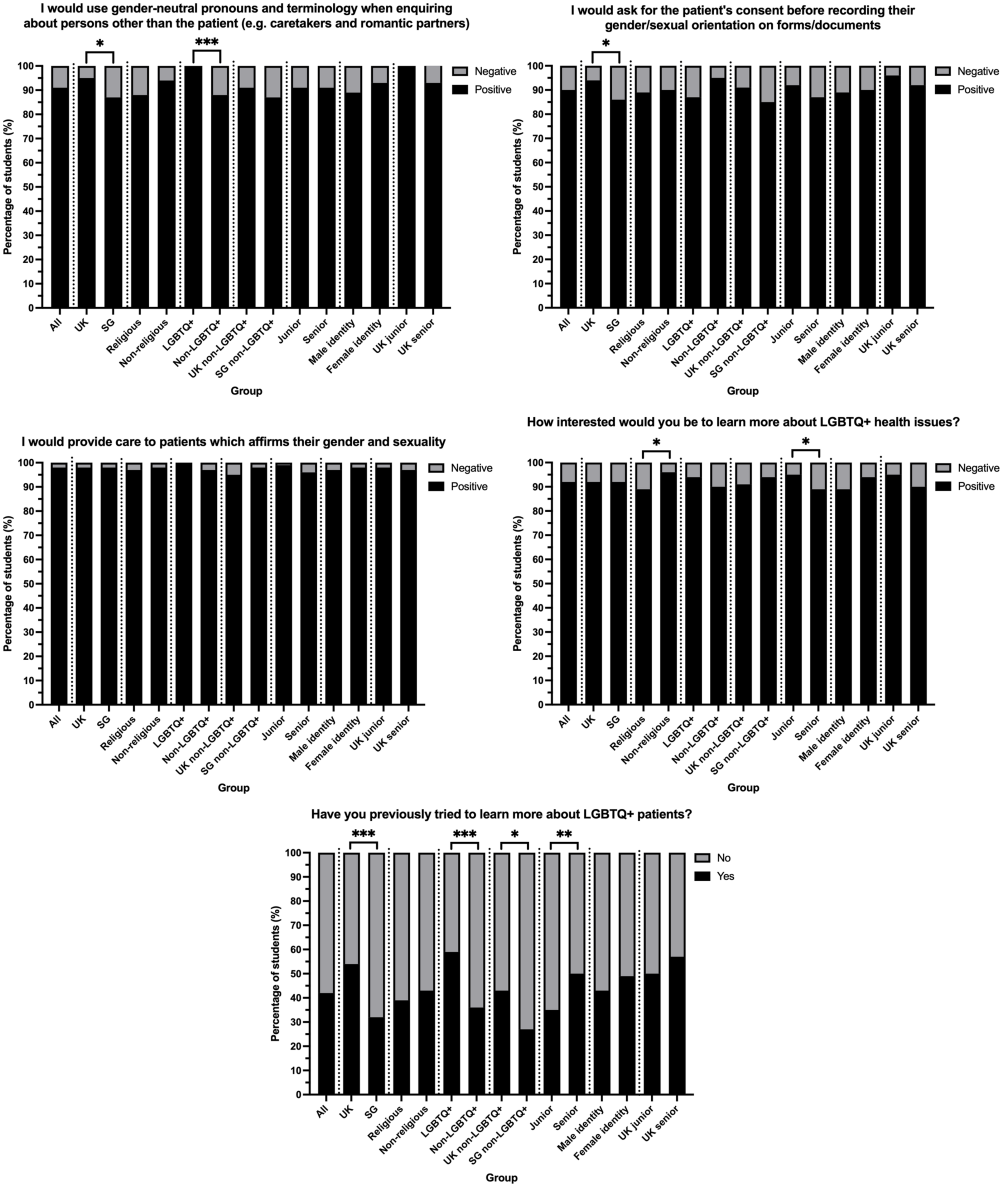
Comparisons between pairs of groups of the percentage of students with positive and negative attitudes towards LGBTQ+ healthcare. * denotes *p* < 0.05, ** denotes *p* < 0.01, *** denotes *p* < 0.001 from Fisher’s exact tests comparing the numbers of students.

Students had positive attitudes towards using gender-neutral pronouns (79% positive), where significantly more UK than SG students (*p* = 0.014) and LGBTQ+ than non-LGBTQ+ students (*p <* 0.001) would use gender-neutral pronouns when enquiring about persons other than the patient. Still, there was no significant difference when comparing non-LGBTQ+ students between the countries (Figure 3). Further, students were 78% positive toward asking patients’ consent before recording their gender/sexual orientation, with no significant difference between the countries when comparing non-LGBTQ+ students and stage of studies. Additionally, 86% of all students wished to provide gender-affirming care, with similar proportions of positive responses between the countries.

When asked how interested students would be in learning more about LGBTQ+ health issues, 92% of respondents were interested or extremely interested in both countries. Non-religious students were more interested than religious students (*p* = 0.035), while more junior than senior students had greater interest (*p* = 0.046). Interest in learning more was not affected by LGBTQ+ identification or by country. 42% of all students had previously tried to learn more about LGBTQ+ patients, with significantly more UK than SG students (*p <* 0.001) and more LGBTQ+ than non-LGBTQ+ students (*p* < 0.001). When considering only non-LGBTQ+ students, more UK than SG students still tried to learn more previously (*p* = 0.033). Moreover, more senior than junior students tried to learn more (*p* = 0.007), but this difference was insignificant (*p* = 0.408) in the UK alone. There were no notable differences across all questions between students who self-identified as male and those who self-identified as female.

### 3.5. Sources of knowledge about LGBTQ+ healthcare

84% of students were negative regarding the adequacy of teaching LGBTQ+ health throughout medical school (Table 2). Significantly more SG than UK respondents that felt they received insufficient teaching surrounding LGBTQ+ health in university modules (91% vs. 77% respectively, *p* = 0.011), clinical settings (89% vs. 74% respectively, *p* = 0.010), and in experiences in interacting with LGBTQ+ patients (88% vs. 68% respectively, *p* = 0.001). Only one respondent across both countries answered ‘Strongly agree’ to the three teaching areas. A more significant proportion of junior than senior UK students were optimistic about the adequacy of education in university modules (18% vs. 4%, *p* = 0.040), but unsurprisingly, senior students had experienced more clinical teachings (*p* = 0.015). Further, junior and senior UK students had similar adequacy of experiences in interacting with LGBTQ+ patients (*p* = 0.076). Junior and senior UK students also had no differences in confidence levels in interacting with LGBTQ+ patients (*p* = 0.692). However, significantly fewer SG than UK students felt confident (51% vs. 19% respectively, *p* < 0.001). Respondents identifying as LGBTQ+ felt much more confident in their interactions (52% positive) than their non-LGBTQ+ identifying colleagues (16% positive; *p* < 0.001).

**Table 2:** Sources of knowledge and training with comparisons between the United Kingdom (UK) and Singapore (SG).

Topic	All respondents (%)	UK respondents (%)	SG respondents (%)	*p*-value (UK vs. SG)
The proportion of students who felt they received insufficient teaching in…				
“University modules”	84	77	91	0.011
“Clinical settings”	82	74	89	0.010
“Interacting with LGBTQ+ patients”	79	68	88	0.001
The proportion of students who did not feel confident in interacting with LGBTQ+ patients				
Do not feel confident	36	19	51	<0.001
Students receiving medical school teaching as a source of learning for these topics				
Blood donation restrictions	32	25	39	0.048
Sexual health	52	44	60	0.034
Gender-related health	14	21	9	0.028
Gender and sexuality-affirming care	8	11	6	0.311
Students receiving clinical placement teaching as a source of learning for these topics				
Blood donation restrictions	11	10	12	0.822
Sexual health	25	27	24	0.746
Gender-related health	6	9	4	0.251
Gender and sexuality-affirming care	4	7	2	0.170
Students learning from sources outside of education for these topics				
Blood donation restrictions	56	72	42	<0.001
Sexual health	63	71	57	0.055
Gender-related health	80	80	79	>0.999
Gender and sexuality-affirming care	32	38	26	0.095
Students did not learn about these topics from within or outside the curriculum				
Blood donation restrictions	25	20	31	0.104
Sexual health	9	11	9	0.814
Gender-related health	14	13	17	0.553
Gender and sexuality-affirming care	63	53	72	0.008

When asked about the sources of learning for various topics, at least 75% of students did not recall receiving teaching from medical school or clinical placements about LGBTQ+ healthcare topics, especially surrounding gender-related health and providing gender-affirming care (89%; Table 2). However, more SG than UK students received teaching surrounding blood donation restrictions (*p =* 0.048) and sexual health (*p =* 0.034), but more UK than SG students received teaching on gender-related health (*p =* 0.028). 80% of students across both countries gathered information about gender-related health from outside formal education. Still, significantly more SG than UK students did not learn about gender and sexuality-affirming care (72% vs. 53%, respectively, *p =* 0.008). For gender-related teaching and providing affirming care, significantly more junior students on the newer curriculum in the UK learnt this than senior students taught via the older curriculum (36% vs. 10% for gender-related teaching, *p* < 0.001; 22% vs. 3% for providing affirming care, *p* < 0.001). However, blood donation restrictions were better learnt by senior UK than junior students (57% vs. 22%, *p* < 0.001), and learning of sexual health content was similar (*p* = 0.093).

### 3.6. Difficulties faced when learning

Qualitative analysis found that many students found it challenging to find information. A lack of resources was mentioned by many students, where LGBTQ+ healthcare was considered a sensitive topic. Students noted the conservative nature of Singaporean society with societal discrimination, where LGBTQ+ individuals were reluctant to communicate their LGBTQ+ identities and religiosity affected stigma.

“Society here is pretty conservative, and there are many Christians who generally have a poor opinion of the LGBTQ+ community, so this combination of factors makes it hard to discuss/learn about these issues in a medical context”. SG student

Difficulties were described in terms of a lack of teaching in the UK and an apparent lack of university interest and investment, which was more evident in senior compared to junior UK students. Students reported difficulty finding reliable information, with information often expressing stigmatising and homophobic attitudes. The information available seemed limited to sexual health.

“Less formal sources of information out there … There’s also just nothing on it in our med school teaching; the only time it was even briefly mentioned was during ID [infectious disease]”. UK senior student

Moreover, UK students did not know how, what, where, and whom to ask for information surrounding LGBTQ+ healthcare. LGBTQ+ and non-LGBTQ+ students voiced similar difficulties when trying to learn more.

### 3.7. Topics students wanted to learn

Qualitative analysis showed further that communication skills were a common topic students wanted to learn, especially regarding how to be sensitive and respectful towards LGBTQ+ patients and how to make LGBTQ+ patients comfortable. Providing inclusive care was mentioned by many UK students, encompassing gender-affirming care and intersectionality. Guidance for patients seeking conversion therapy was mentioned. Symbolism in the form of rainbow badges and lanyards and ways to show support for LGBTQ+ individuals were expressed by many students. Students wanted to learn about barriers and stigmas that the LGBTQ+ communities faced when accessing healthcare and how societal discrimination affected their healthcare.

“I’d like to know more about the experiences of LGBTQ+ patients and what change they’d like to see in healthcare”. UK junior student

Regarding specific healthcare topics, students especially wanted to learn about transgender healthcare, such as hormone therapy and gender reassignment surgery. Gender dysphoria was another key topic. Mental health was commonly mentioned, as well as sexual and reproductive health, including aspects such as fertility and family planning. However, many students were unsure or did not know what topics they wanted to learn.

“Not that I can think of, but I know learning about it is extremely important. I think the medical school should be doing more to dismantle homophobia and transphobia within the student cohort – I know of many people with these beliefs”. UK junior student

### 3.8. Changes to curriculum proposed by students

It was evident from the qualitative analysis that students wanted more teaching and emphasis on LGBTQ+ health issues in their curricula. This was consistent across students, regardless of LGBTQ+ identification.

“More, more, more. The LGBTQ+ community is unfairly disadvantaged everywhere, including in healthcare. There needs to be more awareness, understanding, and knowledge for treating such patients, so they feel comfortable seeking treatment”. UK senior student

Junior students mentioned some sexual health and gender teaching in the newer UK curriculum, whereas senior students perceived a lack of education that junior students did not mention. SG students mentioned no teaching.

“Teaching regarding such issues is inexistent, so a good start would be to implement small modules on the intricacies of caring for LGBTQ+ patients.” SG student

Students in the UK wanted an earlier start to teaching, with sustained regular teaching throughout the curriculum. SG students wanted their medical school to be more open and mentioned the stigma and personal views that medical educators may have and the difficulty of making changes in SG.

“It [teaching] barely exists. I feel that stereotypes and misinformation are rife among my batchmates, and I feel the school should do more to ensure we can nurture doctors who will create a safe space for LGBT patients”. SG student

UK students wanted less heteronormative language used throughout the course and preferred pronouns to be more respected. Students across both schools wanted to be taught by LGBTQ+ individuals or simulated sessions with self-identifying LGBTQ+ patients.

## 4. Discussion

There is a need to expand medical training to incorporate LGBTQ+ healthcare needs better to address the ongoing discrimination in healthcare contexts against these communities. This study contributes to the sparse data on LGBTQ+ medical education in Singapore and the United Kingdom. Overall, knowledge was generally adequate but lacking in certain areas, especially in SG, compared to the UK. Though attitudes towards healthcare needs were positive, our novel qualitative component showed that most students reported low confidence that their medical training sufficiently prepared them to address LGBTQ+ healthcare needs and considered that the inclusion of such content is needed.

### 4.1. Knowledge

Significantly more pronounced in SG than in the UK, it was evident that medical students lacked knowledge surrounding LGBTQ+ healthcare, echoing previous arguments that medical education fails to train and acknowledge future doctors to address LGBTQ+ healthcare inequalities (37). Basic terminology, such as “out [of the closet]” and “men who have sex with men,” were not well understood. Knowledge of healthcare topics such as blood donation, pre-exposure prophylaxis, and HIV prevalence was also lacking. This is important since prior studies found that a lack of knowledge among doctors led to discrimination against LGBTQ+ patients (8, 9). Our findings suggest that this lack of knowledge may have already existed at medical school and that changes in undergraduate curricula may help to reduce discrimination (11). It was apparent that UK students possessed greater knowledge than SG students even when accounting for sexual orientation, perhaps in part, as mentioned by junior UK students qualitatively, the newer curriculum incorporating gender-related teaching. This contrasted with senior students’ responses of a “lack of teaching” and “no teaching” in SG, reinforcing findings from the USA of insufficiency of formal education at medical schools (15). An advantage of this study was that the junior and senior divide in the UK accurately reflected the years of new curriculum changes. UK junior students appreciated the increased university interest and investment in LGBTQ+ healthcare, and they expressed fewer difficulties when trying to learn compared to senior students. However, the lack of difference in knowledge questions answered correctly between UK junior, and senior students may be explained by learning content through clinical years, potentially via real-world experiences with LGBTQ+ patients, that might have counteracted the lack of teaching in their junior years in the older curriculum. Differences in perceptions of LGBTQ+ healthcare between the countries may also be explained by the sociocultural and legal differences between the two countries. Despite this, the differences between the medical schools were not great, with UK respondents answering one more correct question in the knowledge section on average, suggesting that the UK curriculum could be further inclusive, given the much lengthier period since the legal acceptance of LGBTQ+ individuals and the expected ‘excellent care’ of LGBTQ+ patients from their doctors required by the General Medical Council (38).

Religiosity seemed to be linked to lower knowledge levels, corroborating previous European studies (19, 20). The high proportion of religious respondents in SG compared to the UK may contribute to the differences seen between students in the two countries since religious resistance and stigma may negatively affect students’ perceptions of healthcare (21), where religious beliefs that disapprove of same-sex relations present as a frequent motivator to support conversion therapy (39). This may also explain the many SG respondents that would redirect patients enquiring about conversion therapy to specialist agencies despite the lack of evidence supporting this practice (40) and its harm toward patients (41). Although medical schools may have some slight ability to affect physicians-in-training’s heteronormative prejudices (8, 9), shifts in societal attitudes and beliefs towards LGBTQ+ persons are crucially needed, especially in SG. The practices and perspectives of medical students are contextual, and knowledge from a broad array of settings may help to understand and improve the healthcare disparities experienced by LGBTQ+ persons. Many SG students mentioned that religiosity and stigma may affect patients’ willingness to be open to healthcare providers about their sexual orientations, meaning students may not have much exposure to openly self-identifying LGBTQ+ patients, compared to the UK, where students may have more opportunities to do so.

### 4.2. Attitudes

At first, the greater number of UK students having strongly positive attitudes compared to SG suggests that UK students may be more accepting of the LGBTQ+ communities. The difference in attitudes between countries may be attributed to the increased proportion of LGBTQ+ respondents in the UK since attitudes were similar after accounting for LGBTQ+ identification. This finding, although less significant than hypothesised, shows that students are keen to know more and provide affirmative care to LGBTQ+ patients regardless of the sociolegal contexts and the fact that LGBTQ+ care is not specified in the Singapore Medical Council’s outcomes for graduates (42). A similar study found that both non-heterosexuality and religiosity were significant predictors of students’ attitudes (43), corroborating our findings that non-religious students had greater interest in learning more about LGBTQ+ healthcare. A consistent trend across previous studies with medical students was an association between the male gender and negative attitudes (33, 34). Our study found that gender was not associated with negative attitudes, suggesting that gender identity may not affect awareness and interest in LGBTQ+ healthcare issues, and this highly educated group of students are equally motivated to provide affirming care for LGBTQ+ individuals. This suggestion is further supported by free-text responses, which found that students had similar concerns regarding difficulties when trying to learn more regardless of LGBTQ+ identification and voiced similar ideas and expectations for curricula change.

The greater proportion of UK students that would ask for patients’ preferred pronouns than in SG may reflect the lower amount of stigma and increased exposure surrounding the topic in the United Kingdom; indeed, some academic journals have recently adopted including authors’ preferred pronouns (44). Ascertaining this information is vital to mitigate the stigma and discriminatory environment associated with heteronormative perspectives and attitudes among healthcare providers and to nurture healthy doctor-patient relationships that provide optimal outcomes (45, 46).

### 4.3. Sources of knowledge

Medical school training improves knowledge and awareness of LGBTQ+ healthcare issues (47). The barriers to effective curricular materials have previously been mentioned in the literature, including the absence of trained faculty, perceptions from faculty that LGBTQ+ issues are not relevant to the curriculum, content being absent from examinations, and a lack of teaching role models to discuss sexual orientation or gender identity (15, 17, 18, 48). In this study, most students did not feel they had adequate teaching at medical schools surrounding LGBTQ+ patients, which was distinctly more negative in SG than in the UK. Despite the high proportion of LGBTQ+ respondents, this suggests that students want to learn about these topics, but the opportunities are not necessarily present for them to do so.

Many students did not knowingly have interactions with LGBTQ+ patients, supported by the greater proportion of SG than UK students feeling less confident to interact with LGBTQ+ patients, and obtained their information from outside medical education. This finding is not unexpected, given that in the USA, where modest improvements in LGBTQ+ medical education have been made, many medical schools still have no such content in their curricula (49). This may reflect the lack of willingness in SG to discuss LGBTQ+ behaviour and health.

Results suggest that students have some amount of learning about LGBTQ+ sexual health in the senior years of medical school, but there is a significant lack of gender-related teaching in SG. This is corroborated by qualitative data suggesting that LGBTQ+ healthcare education focussed on sexual health, perhaps to the detriment of other important LGBTQ+ healthcare issues such as mental healthcare and healthcare for non-cisgender individuals. Promisingly, more UK and junior respondents learnt about gender-related and gender-affirming topics introduced in the newer curriculum. However, this did not seem to affect students’ knowledge or attitudes towards LGBTQ+ individuals. These results suggest that further changes may be needed in the curriculum. It was outside this study’s scope to formally analyse the effect of the newer curriculum’s coverage of LGBTQ+ inclusive content on students’ perceptions. Nonetheless, hetero- and cis-normative assumptions in the hidden curriculum, which fails to provide equality, diversity, and inclusion for LGBTQ+ people, may still present a concern for medical curricula to address (50).

### 4.4. Future directions

This study indicates that medical schools, especially in SG, could usefully provide more teaching and emphasis on LGBTQ+ healthcare, echoing calls from previous studies (15, 17). As well as being on sexual healthcare, this could also give the students a holistic understanding of the diverse healthcare needs that LGBTQ+ individuals have. Through the qualitative component of our study, students proposed topics they wanted to learn, such as improving communication skills with the LGBTQ+ communities, supporting patients, and providing inclusive care. Our study provides students’ perspectives on changes to the curriculum that medical schools could utilise. Given the potential challenges that both the literature and students in this study expressed (such as lack of trained faculty), temporary interventions may include single lectures (9, 51), online self-administered modules (52), or mixed interventions incorporating didactic lectures, patient groups, and small group sessions (47). To overcome prejudice and biases and improve students’ comfort with LGBTQ+ patients, using additional clinical vignettes and simulations, with the presence of faculty and doctor role models, is also valuable (33, 53, 54), which has been introduced in the UK medical school. Students had further suggestions, including sustained teaching throughout the curriculum and delivery by LGBTQ+ individuals, which corroborated previous findings (55). Future qualitative work in this area would provide further in-depth analyses and better capture students’ perceptions of LGBTQ+ healthcare.

## 5. Conclusion

The foundation for doctor-patient relationships is established during medical school, and healthcare education influences students’ attitudes, knowledge, and skills. This study found slight differences between SG and the UK, where students in the UK were more knowledgeable about LGBTQ+ healthcare, and SG students expressed greater inadequacy in teaching LGBTQ+ healthcare topics. These differences may be attributed to the sociolegal disparities between the countries. There is a need for a review or further review of medical curricula to improve medical students’ training surrounding LGBTQ+ health and create a more equitable healthcare environment, where necessary. Although there may be societal hesitation to include LGBTQ+ health teaching in SG, our study suggests that irrespective of the sociolegal contexts, future doctors want to be equipped with the knowledge and training needed to practice without discrimination and bias.

### 5.1. Limitations

Limitations of the present study include a higher response rate to the survey in SG compared to the UK, which may have affected the interpretations of this study’s results. We cannot know for sure how this disparity should be interpreted. It could mean non-responders and students who did not volunteer to complete the survey were more conservative or less interested in LGBTQ+ healthcare. Alternatively, the differences in response rates may reflect the potential increased appeal of this topic in SG, where LGBTQ+ issues are less openly discussed, and students may be more willing to engage with LGBTQ+ health issues. It could also reflect the increased dissatisfaction of SG students on LGBTQ+ coverage in their curriculum; thus, students perceive themselves as drivers of change. Singaporean national values may also play a part, where service to the community is stressed by the university throughout education. This may mean SG students were more willing to be helpful and collaborative in completing the survey. Finally, our results were only garnered from students in two medical schools, so they may not be generalisable across the entirety of the United Kingdom and Singapore’s medical schools.

## Data availability statement

The raw data supporting the conclusions of this article will be made available by the authors, without undue reservation.

## Ethics statement

The studies involving humans were approved by Imperial College Research Ethics Committee (21IC7342) and Nanyang Technological University Institutional Review Board (IRB-2021-521). The studies were conducted in accordance with the local legislation and institutional requirements. Written informed consent for participation was not required from the participants or the participants’ legal guardians/next of kin in accordance with the national legislation and institutional requirements.

## Author contributions

MF, TZ, RA, XY, SO, AT, SS, and AB contributed to the conception and design of the study. MF, TZ, RA, XY, and SO acquired and analysed data. AB and SS audited the qualitative coding. MF prepared the original draft of the manuscript, with contributions from TZ, RA, SO, and XY. SS, AB, and AT provided supervision and critically revised the manuscript. All authors contributed to the article and approved the submitted version.
