# Supplementary material for: Medical students’ perceptions of LGBTQ+ healthcare in Singapore and the United Kingdom
Source: Front Med (Lausanne). 2023 Oct 24;10:1236715. doi: 10.3389/fmed.2023.1236715 (PMC10627960; doi:10.3389/fmed.2023.1236715)
Supplement: Supplementary file 1 [file Data_Sheet_1.DOCX]

Supplementary Material

Medical Students’ Perceptions of LGBTQ+ Healthcare in Singapore and the United Kingdom

**Michael Xiangchen Fu^1,2^*, Tangming Zou^3^†, Raksha Aiyappan^3^†, Xinyu Ye^1^†, Simisola Onanuga^1^†, Angela Tan^3^, Ana Baptista^1^, Sue Smith^1^**

**†Equal contribution**: These authors contributed equally to this work

*** Correspondence:**Michael Xiangchen Fu
[michael.fu18@imperial.ac.uk](mailto:michael.fu18@imperial.ac.uk); [michael.fu@sjc.ox.ac.uk](mailto:michael.fu@sjc.ox.ac.uk)

# Supplement 1: Survey

## Understanding of LGBTQ+ health issues among medical students

- This **15 minute survey**will assess your understanding of the health needs faced by LGBTQ+ patients.
- We recommend taking this survey **on a mobile device** for a more comfortable user interface.
- We kindly request that you answer these questions **as honestly as possible**, so that we can derive appropriate recommendations toward making healthcare a more inclusive space for all.
- Upon submission of this survey, you will be redirected to a separate form where you may enter the prize draw for a **£10 Amazon voucher** (UK) / **$20 GrabFood voucher** (Singapore) (10 winners per country).

**Please read the participant information sheet before proceeding.**

You may then click "Next" at the bottom of the page.

## Participant information sheet

We would like to invite you to participate in this research project. You should only participate if you want to; choosing not to take part will not disadvantage you in any way. Before you decide whether you want to take part, it is important for you to understand why the research is being done and what your participation will involve. Please take some time to read the following information carefully and discuss it with others if you wish.

**What is the purpose of the study?**

This survey aims to assess the understanding of the health needs of LGBTQ+ patients amongst UK and Singaporean medical students.

**Why am I eligible?**This questionnaire is open to all medical students, including graduate-entry, at Imperial College London, and Nanyang Technological University, as well as those who have graduated earlier this year.
***If you are an external BSc intercalator at Imperial, please exit this survey now***, as unfortunately, we will be unable to include your results in our analysis.

**Do I have to take part?**

It is your decision whether or not to take part. You are under no obligation to do so and you are free to withdraw at any time without giving a reason. If you decide that you would like to take part, you will be asked to give your consent after you have discussed any questions you may still have via the email address jhlgbt@outlook.com

**What do I have to do?**

You can start the questionnaire once you have consented to participating in the study. Submission of a completed questionnaire (by pressing the 'submit' button) implies consent to participate, and for all data collected to be used. There will be no way of retracting this information once submitted, please ensure you are happy with your response before submitting.

After completion of the questionnaire, you will be given a link to a separate online form where you may enter your school email address for a chance to receive a token of thanks (see next section). This is optional, and will not influence your participation in the questionnaire.

**What are the possible benefits of taking part?**

Participation in this study will not necessarily help you personally at this time, although you may see benefits in the future if your medical school makes changes to your curriculum as a result of the findings of this study. We hope that you will find the survey a thought-provoking experience and that you will benefit from sharing your understanding of LGBTQ+ patient specific health needs to help improve medical education and inclusivity in medicine.

You will also be given the chance to win a £10 [GBP] / $20 [SGD] voucher on completion of the questionnaire as a token of thanks for taking part in this study.

**What are the possible risks of taking part?**

We do not anticipate any risks or disadvantages to your taking part. We estimate that the questionnaire should take you no longer than 15 minutes to complete.

**Will my taking part in this study be kept confidential?**

All information which is collected about you during the survey will be anonymised and kept strictly confidential. We will communicate the overall results of the study to the medical schools you are attending, but no data will be attributed to you. We are not collecting names/matriculation number/personal email ID attributable to your responses. The collection of your school email address in a separate form will be solely for alerting you if you have won the voucher and sending it to you, and will not be attached to your survey data where we store it.

We will ask you for some demographic information as we want to find out if there are particular groups of students for whom the education system is not working well. The investigators will be analysing all responses together and will not be going through individual responses. Therefore, any potentially identifiable information will be kept anonymous to the investigators. Only collated results will be potentially published and no individual response will be. It is recommended that you do not include any potentially identifiable information.

If you do not finish the survey for any reason, your results will not be analysed and will be automatically excluded. No data will be stored on the survey platform, the data will only be cached on your own device. If you wish to return, you can continue the form from your previous progress, however, the investigators will not have access to this data.

**What will happen to the results of the research study?**

The findings of the study will be compiled into a report to be circulated internally, to assist Faculties to make changes with recommendations to improve the medical curriculum. The work may also be published in an academic journal. You will not be identified in any report/publication. If you wish, we will provide you with a short summary and a copy of the publication.

**Who is conducting the research?**The project is being led by five medical students, from Imperial College London in the UK and Lee Kong Chian School of Medicine in Singapore, and is supervised by Professor Sue Smith and Dr Angela Tan.

**Who is funding the research?**

Funding is from the Professor Jenny Higham Collaboration Grant at Imperial College London and Lee Kong Chian School of Medicine.

Ethics approval has been obtained for the study from Imperial College London and Lee Kong Chian School of Medicine.

(End of Page 1 )

## Consent

*If you do not consent to participating in the survey, please close the form now.*

**1. By proceeding, I confirm that:**I have read the participant information sheet.

I understand that should I proceed with this survey, the information gathered will be used anonymously and exclusively to assess the level of understanding among medical students of the needs and health issues faced by LGBTQ+ patients.

I consent to the use of information in this manner.

m I have read, understand, and consent

(End of Page 2 )

## Questions about yourself

*This section will collect basic information about yourself. Please answer as accurately as you can.*

**2. What is your age?**

You must be 18 or older to participate in this survey.

____________________

**3. Where do you study?**

m Lee Kong Chian School of Medicine

m Imperial College School of Medicine

This Question is Conditionally Shown if: (3 = Lee Kong Chian School of Medicine)

**4. What is the highest year of study you have completed in medical school?**

m None

m Year 1

m Year 2

m Year 3

m Year 4

m Year 5

This Question is Conditionally Shown if: (3 = Imperial College School of Medicine)

**7. What is the highest year of study you have completed in medical school?** (If you are an external intercalator, please exit this survey, as explained in the participation information sheet)

m None

m Year 1

m Year 2

m Year 3

m Year 4

m Year 5

m Year 6

**10. What is your gender?**

m Female

m Male

m Non-binary

m Other gender

m Prefer not to say

This Question is Conditionally Shown if: (10 = Other gender)

**11. If you prefer to specify your gender, please do so below:**

____________________

**12. What is your religion?**

m Buddhism

m Christianity

m Hinduism

m Islam

m Judaism

m Sikhism

m Other religion

m No religion

m Prefer not to say

This Question is Conditionally Shown if: (12 = Other religion)

**13. If you prefer to specify your religion, please do so below:**

____________________

**15. Do you identify as LGBTQ+?**

m Yes

m No

m Prefer not to say

This Question is Conditionally Shown if: (15 = Yes)

**16. How do you identify?**

Please select all that apply.

q Lesbian

q Gay

q Bisexual

q Transgender

q Queer

q Other identity

q Prefer not to say

This Question is Conditionally Shown if: (15 = Yes)

**17. If you would like to provide additional details regarding your LGBTQ+ identity, please do so below.**

____________________

**18. Do any of your close friends or family members identify as LGBTQ+?**

m Yes

m No

m Prefer not to say

**19. Do you interact on a regular basis with someone who identifies as LGBTQ+?**

m Yes

m No

m Prefer not to say

(End of Page 3 )

## Understanding of LGBTQ+ health issues

*This section will assess your level of understanding of LGBTQ+ health issues.*

- Please read and answer these questions **honestly** and **to the best of your current knowledge**.
- You should choose the option that **best** represents what you believe to be correct, or "**I don't know**" if you think you do not know the correct answer
- At the end of the survey, you will be given access to an **information sheet** where you may find additional details on some of the questions and answers.

*Note: although these questions do not intend to offend or discriminate, some may be of an upsetting nature. If you need to stop participation in the survey, your answers will not be recorded. The information we gather is purely for research purposes so as to gain a broader understanding of the range of views within the 2 cohorts of students. Your participation is greatly appreciated.*

**1. Which of the following figures do you think most accurately reflects the proportion of LGBTQ+ individuals in your country?**

m <0.1%

m 0.1-1%

m 1-10%

m >10%

m I don't know

**2. What is the appropriate term for someone who has revealed their sexual or gender identity to others?**

m Queer

m Open

m Transitioned

m Out [of the closet]

m Passing

m I don't know

**3. The two terms "MSM" and "gay [men]" can be used interchangeably.**

m True

m False

m I don't know

**5. A person who is bisexual:**

m has both female and male sex characteristics.

m experiences attraction to more than one gender.

m identifies as both female and male.

m I don't know

**6. A person who is a trans woman:**

m identifies as female, but was assigned as male at birth.

m identifies as male, but was assigned as female at birth.

m identifies as neither female nor male.

m I don't know

**7. What might the process of gender transitioning possibly involve?**

Please select all that apply, OR "none of the above", OR "I don't know"

q Medical therapy

q Social transitioning

q Legal transitioning

q Religious transitioning

q None of the above

q I don't know

**8. LGBTQ+ individuals can donate blood without any particular restrictions.**

m True

m False

m I don't know

**9. The LGBTQ+ community has a higher incidence and prevalence of HIV/AIDS.**

m True

m False

m I don't know

**10. What is the difference between PrEP and PEP?**

m PrEP is taken before having sex; PEP is taken after having sex

m PEP is taken before having sex; PrEP is taken after having sex

m PrEP and PEP are both taken before having sex

m PrEP and PEP are both taken after having sex

m PEP is only meant for needle stick injury and is unrelated to PrEP

m I don't know

**11. Homosexuality is a psychological condition that may be treated and cured.**

m True

m False

m I don't know

**12. What is gender dysphoria?**

m Gender dysphoria is a term that describes a sense of unease that a person may have because of a mismatch between their biological sex and their gender identity.

m Gender dysphoria is a term that describes a sense of unease that a person may have because of society's perceptions toward their gender.

m I don't know

**13. You are about to see a patient in the clinic. The patient’s medical record indicates “Female”. When the patient enters, you notice that the patient presents as masculine. Which pronouns would you use to refer to the patient when writing in the notes?**

m "She/her"

m "He/him"

m "They/them"

m Ask if the patient is transgender

m Ask for the patient's preferred pronouns

**14. In the event of a patient enquiring about or requesting conversion therapy for themself, which of these options would align with your likely course of action? (Choose all that apply)**

q Explain to them that conversion therapy does not have enough scientific evidence to support it

q Assist your patient in understanding more about various sexual orientations

q Redirect them to conversion therapy agencies

q Redirect them to organisations providing support services for LGBTQ+ individuals

**15. How far would you accommodate the following concerns in your clinical practice?**

|  | Strongly disagree | Disagree | Neither agree nor disagree | Agree | Strongly agree |
| --- | --- | --- | --- | --- | --- |
| **I would use gender-neutral pronouns and terminology when enquiring about persons other than the patient (e.g. caretakers and romantic partners)** | m | m | m | m | m |
| **I would ask for the patient's consent before recording their gender/sexual orientation on forms/documents** | m | m | m | m | m |
| **I would provide care to patients which affirms their gender and sexuality** | m | m | m | m | m |

(End of Page 4 )

## School-based teaching of LGBTQ+ health issues

*This section will evaluate the teaching you may have received regarding LGBTQ+ health issues.*

**17. How would you rate your learning regarding LGBTQ+ patients?**

|  | Strongly disagree | Disagree | Neither agree nor disagree | Agree | Strongly agree |
| --- | --- | --- | --- | --- | --- |
| **I have received adequate teaching about LGBTQ+ patients in university-based modules/blocks** | m | m | m | m | m |
| **I have received adequate teaching about LGBTQ+ patients in clinical settings** | m | m | m | m | m |
| **I have had ample experience in interacting with LGBTQ+ patients in a clinical setting** | m | m | m | m | m |
| **I felt confident in interactions with LGBTQ+ patients** | m | m | m | m | m |

## Sources of knowledge about LGBTQ+ issues

*This section will assess where you may have learned about issues relating to the LGBTQ+ community and LGBTQ+ health.*

**18. How have you learned about LGBTQ+ issues in general?***(e.g. what LGBTQ+ stands for, or what "in the closet"/"out of the closet" refers to)*

Please select all that apply.

q Social media (e.g. Instagram, Facebook)

q Other digital media (e.g. Wikipedia)

q Traditional media (print, news, television)

q Organisations working with the LGBTQ+ community

q Religious organisations

q School

q Work

q Friends/family/peers who identify as LGBTQ+

q Friends/family/peers who do not identify as LGBTQ+

q I have not learned about these

**19. How have you learned about the following topics?**

Please select all that apply.

|  | Medical school teaching which all students receive | Clinical placement/postings | Enrichment or elective programs | Outside of education (e.g. friends, family, media) | I have not learned about this |
| --- | --- | --- | --- | --- | --- |
| **Blood donation restrictions** | q | q | q | q | q |
| **Sexual health (STIs, HIV/AIDS, practice of safe sex, and PrEP)** | q | q | q | q | q |
| **Gender (knowledge of gender, gender dysphoria, gender transition/reassignment)** | q | q | q | q | q |
| **How to provide gender and sexuality affirming care** | q | q | q | q | q |

## Further learning about LGBTQ+ issues

*This section will gauge your opinions on if and where your education in LGBTQ+ health could be improved.*

**21. How interested would you be to learn more about LGBTQ+ health issues?**

m Extremely interested

m Interested

m Uninterested

m Extremely uninterested

**22. Have you previously tried to learn more about LGBTQ+ patients?**

m Yes

m No

This Question is Conditionally Shown if: (22 = Yes)

**23. Have you encountered any difficulties when trying to learn more about LGBTQ+ patients?**

m Yes

m No

This Question is Conditionally Shown if: (22 = Yes AND23 = Yes)

**24. What difficulties did you face?**

____________________

**25. Are there any LGBTQ+ healthcare-related topics you would like to learn more about?**

____________________

**26. Are there any changes you would like to see in current formal teaching regarding LGBTQ+ health issues?**

____________________

(End of Page 5 )

# To complete the survey, please click "Submit Survey".

You will be redirected to a separate **post-survey form**, where you will find:

- A link to an **information sheet** if you would like to learn more about the topics explored in this survey.
- An email entry for the **prize draw** for ten **£10 Amazon vouchers** (UK) / ten **$20 GrabFood vouchers** (SG).

**46. Do you have any queries for our team / any thoughts you would like to share with us regarding this survey?**

____________________

(End of Page 6 )
